# Risks and neurological benefits of meningioma surgery in elderly patients compared to young patients

**DOI:** 10.1007/s11060-021-03832-5

**Published:** 2021-09-01

**Authors:** Hajrullah Ahmeti, Christoph Borzikowsky, Dieter Hollander, Christoph Röcken, Olav Jansen, Michael Synowitz, Maximilian H. Mehdorn

**Affiliations:** 1 Department of Neurosurgery, University Hospital Schleswig-Holstein, Campus Kiel, Arnold-Heller-Str. 3, 24105 Kiel, Germany; 2 Institute of Medical Informatics und Statistics, University Hospital Schleswig-Holstein, Kiel University, Kiel, Germany; 3 Department of Anesthesiology, University Hospital Schleswig-Holstein, Campus Kiel, Kiel, Germany; 4 Department of Pathology, University Hospital Schleswig-Holstein, Campus Kiel, Kiel, Germany; 5 Department of Radiology and Neuroradiology, University Hospital Schleswig-Holstein, Campus Kiel, Kiel, Germany

**Keywords:** Elderly patients, Comorbidities, Intracranial meningiomas, Operative risks, Meningioma surgery, Neurological conditions

## Abstract

**Introduction:**

While surgery is the primary treatment choice for intracranial meningiomas in young patients, surgery in elderly patients, especially those with pre-existing comorbidities, has been the subject of repeated discussion. This study investigated the postoperative risks and neurological benefits of meningioma surgery in elderly patients compared to young patients.

**Methods:**

In total, 768 patients were included and divided into two main groups: group I (age: ≤ 64 years; 484 young patients) and group II (age: ≥ 65 years; 284 elderly patients). Group II was subdivided into: IIa (age: 65–69 years), IIb (age: 70–79 years); and IIc (age: ≥ 80 years).

**Results:**

The total tumor resection rate was higher in the elderly cohort than in the young cohort (84.5 and 76.2%, respectively). 154 young patients (31.8%) and 132 elderly patients (46.5%) developed postoperative morbidities, with the three most common being bleeding (12.9%), cranial nerve disorder (10%) and CSF fistula (8.1%). Postoperative bleeding, palsy, speech disorder, pneumonia and renal insufficiency were dependent on age (r = 0.123, p = 0.001; r = 0.089, p = 0.014; r = 0.100, p = 0.006; r = 0.098, p = 0.007 and r = 0.084, p = 0.020) and presented more often in elderly patients. 6 young and 15 elderly patients died during the 17.4-year observation period. Most patients showed a significant improvement in postoperative KPS (p < 0.001), except those over 80 years old (p = 0.753). The KPS at the last follow-up was significantly improved in all patients (p < 0.001).

**Conclusion:**

Meningioma surgery is associated with a higher rate of postoperative complications in elderly patients than in young patients. Most elderly patients, similar to young patients, show a significant improvement in neurological status postoperatively.

**Supplementary Information:**

The online version contains supplementary material available at 10.1007/s11060-021-03832-5.

## Introduction

Meningiomas are the most common primary brain tumors in adults. According to the Central Brain Tumor Registry of the United States of America (CBTRUS), meningiomas account for 38.3% of all primary brain tumors [1]. The treatment of choice in meningiomas is surgery [2]. It is well known that meningiomas are more common in elderly patients than in young patients [2–4]. The incidence rate of intracranial meningiomas increases dramatically with age. Patients between the ages of 20 and 34 years have an incidence rate for meningiomas of 1.46/100,000. This number increases to 29.08/100,000 in patients between the ages of 65 and 74 years and to 55.08/100,000 in patients over 85 years old [1]. In particular, elderly patients often have high rates of comorbidities, which suggests a high perioperative risk. Increased morbidity and mortality after meningioma surgery in elderly patients have been reported in many studies [2, 5–8]. Therefore, a ‘wait and see’ strategy, especially in patients with comorbidities and few symptomatic tumors, might be preferred. However, Arienta et al. showed a significantly increased tumor-related risk of morbidity and mortality in their ‘wait and see’ group [9]. In addition, atypical and anaplastic meningiomas, which behave more aggressively than benign meningiomas, seem to occur more often in elderly patients and could complicate the clinical course [1, 2, 10].

Most presented patient series on this subject have a small number of elderly patients. In the present study, with a large representative series, we evaluated the postoperative risk and neurological benefits of meningioma surgery in elderly patients in different age groups compared to young patients.

## Methods

In this retrospective study, all patients who underwent primary surgery for intracranial meningioma in our department between 2003 and 2019 were included. Patients who received conservative therapy and those with recurrent meningiomas were excluded from the study. The patients were divided into two groups: group I (aged ≤ 64 years) and group II (aged ≥ 65 years). Furthermore, group II was divided into three subgroups: IIa (aged 65–69 years); IIb aged 70–79 years) and IIc (aged over 80 years). Group I was regarded as the control group (young patients).

The patients were operated on by different neurosurgeons with the intention of maximal safe feasible tumor resection. All patients were monitored postoperatively in our neurosurgical intensive care unit and were examined neurologically by a neurosurgeon. The diagnosis of meningioma was confirmed histologically according to WHO criteria [11, 12].

All patient’s demographics and characteristics, comorbidities, tumor and treatment characteristics, neurological status according to the KPS [14] preoperatively, postoperatively and at the time of last follow-up, ASA score according to the American Society of Anesthesiologists score [13], postoperative complications within 30 days and new neurological deficits, and follow-up data were collected by using medical inpatient and outpatient records of our department. Depending on the size, site and vascularization, tumor complexity was classified as uncritical, moderately critical and critical by an experienced neurosurgeon.

## Statistics

All statistical analyses were carried out with IBM SPSS Statistics for Windows [15]. The following statistical analyses have been conducted: Absolute and relative frequencies, mean and standard deviation (SD), median and range of values, Spearman’s rank correlation coefficients, Chi-squared (χ^2^), Fisher’s exact tests, two-sample t-tests, multiple logistic and multiple linear regression analyses, unstandardized and standardized regression coefficients, 95% confidence intervals, p-values and odds ratios (ORs). p-Values < 0.05 were regarded as statistically significant.

## Results

### General characteristics

In this study, 768 patients (484 young and 284 elderly patients) were included (Table 1). The three most common tumor sites were convexity (28.8%), sphenoid ridge (20.1%) and falx/parasagittal (15.2%). In all, 62 patients (8.1%) had multilocular meningiomas as follows: 37 patients (7.6%) in group I and 25 patients (8.8%) in group II, namely. The extent of edema showed a significant correlation with the size of the tumor in young (group I) and elderly patients (group II), and the larger the tumor was, the more extensive the edema (group I: r = 0.468, p < 0.001; group II: r = 0.410, p < 0.001). In the subgroups, only subgroup IIc did not show a correlation between tumor size and edema (IIa: r = 0.623, p < 0.001; IIb: r = 0.333, p = 0.001; IIc: r = 0.101, p = 0.576).

**Table 1:** General patient characteristics

General characteristics	All patients		Group I (age: ≤ 64 yrs)		Group II (age: ≥ 65 yrs)		Subgroup IIa (age: 65–69 yrs)		Subgroup IIb (age: 70–79 yrs)		Subgroup IIc (age: ≥ 80 yrs)	
	No.	%	No.	%	No.	%	No.	%	No.	%	No.	%
No. of patients	768	100	484	63	284	37	101	13.1	142	18.7	41	5.3
Age (median)	60	–	53	–	72	–	67	–	73	–	83	–
Sex												
Female	579	75.4	376	77.7	203	71.5	70	69.3	102	71.5	31	75.6
Male	189	24.6	108	22.3	81	28.5	31	30.7	40	28.5	10	24.4
Tumor size												
1–3 cm	252	32.8	170	35.1	82	28.9	35	34.7	36	25	11	26.8
3–6 cm	233	30.3	137	28.3	96	33.8	24	23.8	57	39.6	16	39
6–10 cm	61	7.9	37	7.6	24	8.5	6	5.9	12	8.3	6	14.6
10–15 cm	3	0.4	2	0.4	1	0.4	–	–	1	0.7	–	–
> 20 cm	1	0.1	–	–	1	0.4	–	–	–	–	1	2.4
NK	218	28.4	138	28.5	80	28.2	36	35.6	38	26.4	7	17.1
Brain edema												
None	380	49.5	259	53.5	121	42.6	53	52.5	62	43.1	7	17.1
Moderate	190	24.7	113	23.3	77	27.1	20	19.8	39	27.1	19	46.3
Large	148	19.3	75	15.5	73	25.7	20	19.8	39	27.1	14	34.1
NK	50	6.5	37	7.6	13	4.6	8	7.9	4	2.8	1	2.4
Asymptomatic meningioma	73	9.5	50	10.3	23	8.1	11	10.9	13	9	–	–

*NK* not known, *yrs* years

### Symptoms, neurological conditions and comorbidities

The young patients more often suffered than elderly patients from cranial nerve disorder (nerve II-XII) (p < 0.001) and headache (p < 0.001). On the other hand, the elderly patients more often had gait disorders and dizziness (p < 0.001) and personality changes and memory disorders (p < 0.001; Table 2). The larger the tumor was, the more frequently personality change and memory disorder (r = 0.228, p < 0.001), palsy (r = 0.201, p < 0.001), speech disorder (r = 0.146, p = 0.001), seizure (r = 0.096, p = 0.024), olfactory dysfunction (r = 0.089, p = 0.038) and cranial nerve disorder (nerve II-XII) (r = 0.097, p = 0.023) occurred.

**Table 2:** Symptoms, neurological conditions, comorbidities and ASA score

Symptoms/neurological conditions and comorbidities	All patients		Group I (age: ≤ 64 yrs)		Group II (age: ≥ 65 yrs)		Subgroup IIa (age: 65–69 yrs)		Subgroup IIb (age: 70–79 yrs)		Subgroup IIc (age: ≥ 80 yrs)	
	No.	%	No.	%	No.	%	No.	%	No.	%	No.	%
Symptoms/neurological conditions												
Cranial nerve disorder	237	30.9	171	35.3	66	23.2	25	24.8	30	20.8	11	26.8
Headache	229	29.8	168	34.7	61	21.5	26	25.7	31	21.5	4	9.8
Gait disorder and dizziness	148	19.3	73	15.1	75	26.4	24	23.8	37	25.7	15	36.6
Seizure	106	13.8	66	13.6	40	14.1	10	9.9	20	13.9	10	24.4
Personality change and memory disorder	104	13.5	49	10.1	55	19.4	17	16.8	27	18.8	11	26.8
Palsy	62	8.1	28	5.8	34	12	5	5	21	14.6	8	19.5
Sensitive disorder	45	5.9	29	6	16	5.6	4	4	11	7.6	1	2.4
Speech disorder	43	5.6	20	4.1	23	8.1	9	8.6	8	5.6	6	14.6
Swelling	34	4.4	30	6.2	4	1.4	1	1	3	2.1	–	–
Olfactory dysfunction	28	3.6	17	3.5	11	3.9	6	5.9	5	3.5	–	–
Hormonal disorder	5	0.7	5	1	–	–	–	–	–	–	–	–
Comorbidities												
Arterial hypertension	323	42.1	147	30.4	176	62	48	47.5	97	67.4	33	80.5
Heart failure/arrhythmia	74	9.6	18	3.7	56	19.7	12	11.9	30	20.8	14	34.1
Heart anomaly	23	3	9	1.9	14	4.9	4	4	6	4.2	4	9.8
Diabetes mellitus	74	9.6	31	6.4	43	15.1	13	12.9	26	18.1	5	12.2
COPD/asthma	48	6.3	21	4.3	27	9.5	4	4	19	13.2	4	9.8
Any cancers	115	15	60	12.4	55	19.4	13	12.9	30	20.8	12	29.3
Renal failure	23	3	8	1.7	15	5.3	2	2	9	6.3	4	9.8
Endocrine disease	120	15.6	73	15.1	47	16.5	10	9.9	30	20.8	8	19.5
Depression	32	4.2	28	5.8	4	1.4	2	2	2	1.4	–	–
Neurosurgical intervention	32	4.2	19	3.9	13	4.6	7	6.9	4	2.8	2	4.9
Other diseases^a^	286	37.2	147	30.4	139	48.9	42	41.6	72	50	26	63.4
No comorbidity	210	27.3	173	35.7	37	13	20	19.8	16	11.1	1	2.4
Anticoagulant	102	13.3	26	5.4	76	26.8	20	19.8	44	30.6	13	32.7
ASA score												
1	114	14.8	95	19.6	19	6.7	7	6.9	12	8.3	–	–
2	435	56.6	304	62.8	131	46.1	57	56.4	59	41	17	41.5
3	191	24.9	69	14.3	122	43	35	34.7	66	45.8	21	51.2
4	16	2.1	8	1.7	8	2.8	–	–	5	3.5	3	7.3
5	2	0.3	2	0.4	–	–	–	–	–	–	–	–

*yrs* years

^a^Other diseases represent different diseases, such as allergies, peripheral arterial disease, musculoskeletal diseases and different surgical interventions

**Fig. 1:**
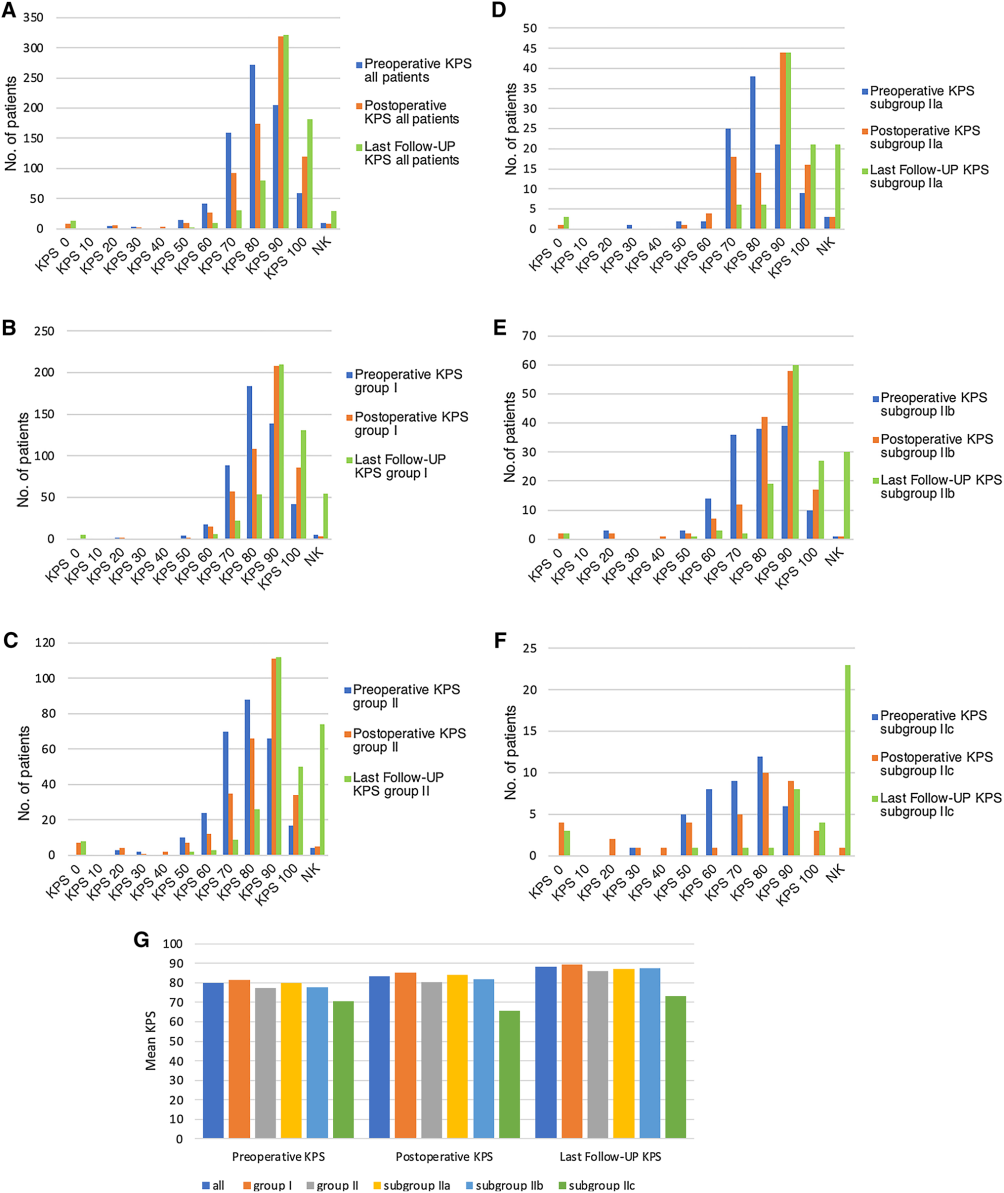
Preoperative, postoperative and last follow-up Karnofsky Performance Status Scale (KPS) (**A**–**G**). *NK* not known

The mean preoperative KPS was 79.90 SD ± 12.31 (range 20–100) in the entire cohort. The young patients showed a mean preoperative KPS of 81.44 (SD ± 11.11), and the elderly patients showed a mean preoperative KPS of 77.28 (SD ± 13.74); the results for subgroups IIa, IIb, and IIc were 79.89 (SD ± 11.70), 77.76 (SD ± 14.50) and 70.48 (SD ± 14.13), respectively (Fig. 1; Supplemental Content).

Thirty-two patients (4.2%) had different neurosurgical interventions in their medical history, most often spine surgery. With the exception of depression, which was more common in the young patients (group I vs. II: 28 patients (5.8%) vs. 4 patients (1.4%), (p = 0.002)), all other comorbidities showed an increasing incidence with age (Table 2).

### Surgery, histology, postoperative morbidity, and mortality

Total tumor resection was achieved in 609 patients (79.3%; Table 3). In the young patients but not in the elderly patients (group II, p = 0.345), the extent of tumor resection was significantly dependent on tumor size (group I: r = 0.148, p = 0.007). Postoperatively, 154 patients (31.8%) in the young cohort and 132 patients (46.5%) in the elderly cohort developed morbidities (Table 3). Postoperative bleeding, palsy, speech disorder, pneumonia and renal insufficiency depended on age (r = 0.123, p = 0.001; r = 0.089, p = 0.014; r = 0.100, p = 0.006; r = 0.098, p = 0.007 and r = 0.084, p = 0.020) and were presented more often in elderly patients (Table 3). In elderly patients, males were more frequently affected by bleeding than females (r = 0.122, p = 0.001).

**Table 3:** Surgery, histology, postoperative morbidity, mean and maximum follow-up

Surgery, histology, postoperative morbidity, follow-up	All patients		Group I (age: ≤ 64 yrs)		Group II (age: ≥ 65 yrs)		Subgroup IIa (age: 65–69 yrs)		Subgroup IIb (age: 70–79 yrs)		Subgroup IIc (age: ≥ 80 yrs)	
	No.	%	No.	%	No.	%	No.	%	No.	%	No.	%
Simpson grade												
I	391	50.9	233	48.1	158	55.6	54	53.5	84	58.3	20	48.8
II	218	28.4	136	28.1	82	28.9	35	34.7	35	24.3	13	31.7
III	28	3.6	21	4.3	7	2.5	3	3	3	2.1	1	2.4
IV	86	11.2	62	12.8	24	8.5	6	5.9	14	9.7	5	12.2
V	7	0.9	6	1.2	1	0.4	–	–	1	0.7	–	–
NK	38	4.9	26	5.4	12	4.2	3	3	7	4.9	2	4.9
WHO classification												
I	673	87.6	423	87.4	250	88	89	88.1	131	91	32	78
II	67	8.7	46	9.5	21	7.4	9	8.9	6	4.2	6	14.6
III	10	1.3	5	1	5	1.8	1	1	3	2.1	1	2.4
NK	7	0.9	2	0.4	5	1.8	1	1	2	1.4	2	4.9
Mean Ki67-index	5.8		5.6		6		5.1		5.9		8.2	
Entire postoperative morbidity	286	37.2	154	31.8	132	46.5	45	44.6	66	45.8	21	51.2
Postoperative morbidities												
Bleeding	99	12.9	44	9.1	55	19.4	16	15.8	27	18.8	12	29.3
Palsy	25	3.3	8	1.7	17	6	6	5.9	8	5.6	3	7.3
Cranial nerve disorder	77	10	49	10.1	28	9.9	11	10.9	15	10.4	2	4.9
CFS fistula	62	8.1	37	7.6	25	8.8	11	10.9	12	8.3	2	4.9
Speech disorder	20	2.6	5	1	15	5.3	6	5.9	6	4.2	3	7.3
Seizure	29	3.8	14	2.9	15	5.3	5	5	5	3.5	5	12.2
Meningitis	4	0.5	3	0.6	1	0.4	–	–	1	0.7	–	–
Delirium	14	1.8	4	0.8	10	3.5	3	3	6	4.2	1	2.4
Hormonal disorder	4	0.5	4	0.8	–	–	–	–	–	–	–	–
Ischemic infraction	14	1.8	6	1.2	8	2.8	2	2	5	3.5	1	2.4
Vein thrombosis	8	1	6	1.2	2	0.7	–	–	2	1.4	–	–
Pulmonary embolism	4	0.5	1	0.2	3	1.1	–	–	2	1.4	1	2.4
Pneumonia	11	1.4	3	0.6	8	2.8	1	1	4	2.8	3	7.3
Iatrogenic pneumothorax	11	1.4	6	1.2	5	1.8	1	1	3	2.1	1	2.4
Respiratory insufficiency	7	0.9	3	0.6	4	1.4	–	–	2	1.4	2	4.9
Renal insufficiency	2	0.3	–	–	2	0.7	–	–	–	–	2	4.9
Heart attack	1	0.1	–	–	1	0.4	–	–	1	0.7	–	–
Heart/circulatory failure	7	0.9	3	0.6	4	1.4	–	–	–	–	4	2.8
Discharge to home	586	76.3	394	81.4	192	67.6	77	76.2	98	68.1	18	43.9
Discharge to rehabilitation clinic or another department	141	18.4	61	12.6	80	28.2	15	14.9	43	29.9	22	53.7
Mean follow-up in months	40		47		28		36		26		8	
Maximum follow-up in months	215		215		155		202		132		38	

*NK* not known, *yrs* years

Tumor size correlated with the occurrence of bleeding (r = 0.186, p < 0.001), seizures (r = 0.133, p = 0.002), CSF fistulas (r = 0.95, p = 0.027), delirium (r = 0.102, p = 0.017) and pneumonia (r = 0.122, p = 0.004). In addition, the complexity of the tumor also correlated with the risk of bleeding (r = 0.136, p < 0.001), cranial nerve disorder (r = 0.165, p < 0.001) and CSF fistula (r = 0.132, p < 0.001) in young and elderly patients. Tumor site did not have any effect on postoperative morbidity or mortality. On the other hand, the extent of edema indicated a significant risk for bleeding (r = 0.165, p < 0.001), palsy (r = 0.076, p = 0.042), cranial nerve disorder (r = 0.101, p = 0.007), ischemic infarction (r = 0.110, p = 0.003), delirium (r = 0.108, p = 0.004), speech disorder (r = 0.103, p = 0.006) and pneumonia (r = 0.076, p = 0.041). The extent of tumor resection showed a significant effect on the occurrence of cranial nerve disorder in the young cohort (r = 0.186, p < 0.001) and in subgroup IIb (r = 0.178, p = 0.38). Some particular postoperative morbidities were more frequently observed when the following comorbidities were present: arterial hypertension, heart failure/arrhythmia, renal failure and depression. Arterial hypertension and heart failure/arrhythmia showed a high risk for seizures (r = 0.094, p = 0.009; r = 0.122, p = 0.001) and speech disorder (r = 0.109, p = 0.003; r = 0.113, p = 0.002) and depression for respiratory failure (r = 0.117, p = 0.001). Additionally, heart failure/arrhythmia indicated a significantly higher incidence of bleeding (r = 0.190, p < 0.001). An increased risk for developing postoperative bleeding in patients treated with perioperative anticoagulant drugs was observed in the entire cohort (r = 0.089, p = 0.013) but not in the young (group I: r = 0.012, p = 0.792) or elderly cohorts (group II: r = 0.086, p = 0.147).

Multiple logistic regression analysis showed a significant impact of brain edema (OR = 2.34, 95% CI [1.36; 4.03], p = 0.002), sex (OR = 0.30, 95% CI [0.12; 0.72], p = 0.008) and WHO classification of tumors (OR = 2.82, 95% CI [1.17; 6.76], p = 0.020) on postoperative bleeding in elderly patients and of tumor size (OR = 2.09, 95% CI [1.06; 4.10], p = 0.032) on postoperative bleeding in young patients. The extent of tumor resection (OR = 2.01, 95% CI [1.22; 3.31], p = 0.006) in elderly patients and the complexity of the tumor (OR = 2.43, 95% CI [1.16; 5.07], p = 0.018) and brain edema (OR = 0.5, 95% CI [0.27; 0.93], p = 0.029) in young patients had a significant influence on postoperative cranial nerve (II-XII) disorder. CSF fistula was significantly affected by the complexity of the tumor (OR = 11.63, 95% CI [1.24; 109.01], p = 0.032) in elderly patients. Postoperative seizures were significantly influenced by tumor size (OR = 4.28, 95% CI [1.76; 10.38], p = 0.001) in elderly patients and by the complexity of the tumor (OR = 0.25, 95% CI [0.07; 0.89], p = 0.032) in young patients.

The mean postoperative KPS was 83.47 (SD ± 15.46) (range 0–100) in the entire cohort. The young patients had a mean postoperative KPS of 85.36 (SD ± 12.29), and of the elderly patients, the mean postoperative KPS was 80.21 (SD ± 19.35). With increasing age, the mean postoperative KPS was lower (subgroup: IIa 83.98 (SD ± 14.34), IIb 81.95 (SD ± 16.66) and IIc 65.75 (SD ± 30.11)) (Fig. 1; Supplemental Content). Advancing age had a significant effect on postoperative KPS only in patients over 80 years old (p = 0.004). The entire cohort, groups I and II and subgroups IIa and IIb showed a significant improvement in the postoperative KPS compared to the preoperative KPS (p < 0.001); in contrast, subgroup IIc showed a deterioration of postoperative KPS.

At the time of the last follow-up, all patients showed a significant improvement in KPS (entire cohort: 88.15 (SD ± 15.60); group I: 89.18 (SD ± 13.27); group II: 86.04 (SD ± 19.39); subgroups: IIa 87 (SD ± 19.05), IIb 87.63 (SD ± 14.95) and IIc 73.33 (SD ± 35.81)), also compared to the postoperative KPS (p < 0.001; Fig. 1; Supplemental Content). At the time of the last follow-up, 54 patients showed deterioration in KPS compared to the postoperative KPS (26 patients (3.4%) tumor-related; 15 patients (2%) tumor-unrelated; 13 patients (1.7%) by pain or hypoesthesia in the surgical scar area).

The postoperative KPS and the KPS at the last follow-up correlated significantly with the preoperative KPS (r = 0.681, p < 0.001 and r = 0.346, p < 0.001). The extent of tumor resection affected the postoperative KPS and the KPS at the last follow-up only in the young cohort; the higher the resection rate was, the higher the postoperative KPS and the KPS at the last follow-up (group I: r = 0.225, p < 0.001 and r = 0.243, p < 0.001). The postoperative KPS was also dependent on tumor size only in the young cohort (group I: r = 0.207, p < 0.001). On the other hand, tumor complexity and brain edema negatively influenced the postoperative KPS in young patients (group I: r = 0.335, p < 0.001 and r = 0.147, p = 0.002) and elderly patients (group II: r = 0.246, p = 0.001 and r = 0.191, p = 0.002). The KPS at the last follow-up was negatively affected by tumor complexity but not by brain edema (group I: r = 0.183, p < 0.001; r = 0.012, p = 0.812 and group II: r = 0.175, p = 0.012; r = 0.092, p < 0.193).

The multiple linear regression analysis showed a significantly importance of preoperative KPS (b = 0.66, 95% CI [0.47; 0.84], β = 0.45, p < 0.001) and age (b = − 0.49, 95% CI [– 0.89; – 0.09], β = – 0.15, p < 0.016) on postoperative KPS in elderly patients, and of preoperative KPS (b = 0.49, 95% CI [0.41; 0.57], β = 0.54, p < 0.001), extent of tumor resection (b = – 1.63, 95% CI [– 2.52; – 0.74], β = – 0.17, p < 0.001), ASA score (b = – 1.69, 95% CI [– 2.94; – 0.43], β = – 0.11, p = 0.008) and complexity of the tumor (b = – 1.62, 95% CI [– 2.95; – 0.28], β = – 0.11, p < 0.017) on postoperative KPS in young patients. The KPS at the last follow-up was significantly affected only by the ASA score (b = – 8.06, 95% CI [– 12.60; – 3.53], β = – 0.28, p = 0.001) in elderly patients and by the preoperative KPS (b = 0.3, 95% CI [0.17; 0.44], β = 0.25, p < 0.001) and extent of tumor resection (b = – 2.17, 95% CI [– 3.52; – 0.82], β = – 0.17, p = 0.002) in young patients.

Twenty-one patients (2.7%) died in an observation period of 17.4 years. Ten patients (1.3%: 3 (0.6%) young and 7 (2.5%) elderly patients) experienced tumor-related death, and 11 patients (1.4%: 3 (0.6%) young and 8 (2.8%) elderly patients) experienced non-tumor-related death. The highest mortality rate was in patients over 80 years of age (7 patients (17%); Table 4).

**Table 4:** Deceased patients

Patient	Age (years)	Sex	Tumor site	Preoperative comorbidity	ASA score	Cause of death	Time of death
1	86	f	Falx	Arterial hypertension, heart arrhythmia, anticoagulant drug	3	Bleeding	4 days
2	92	f	Sphenoid ridge	Renal insufficiency	3	Multiple organ failure	4 days
3	79	m	Sphenoid ridge	Arterial hypertension, diabetes mellitus, cancer	4	Subarachnoidal hemorrhage (incidental meningioma)	10 days
4	88	f	Falx	Arterial hypertension, heart insufficiency, anticoagulant drug	3	seizure	12 days
5	80	m	Falx	Arterial hypertension, heart insufficiency, anticoagulant drug	2	Bleeding, multiple organ failure	14 days
6	72	m	Falx	Arterial hypertension, heart attack, heart insufficiency, COPD	3	Heart failure pneumonia	15 days
7	66	m	Sphenoid ridge	Arterial hypertension, heart attack, peripheral arterial disease, anticoagulant drug	3	Bleeding	20 days
8	45	f	Sphenoid ridge	−	2	Bleeding	23 days
9	76	f	Convexity	Arterial hypertension, heart insufficiency, COPD, depression, renal cell carcinoma	3	Pneumonia and heart insufficiency	38 days
10	86	f	Tentorium	−	2	Recurrence of atypical meningioma with intracerebral bleeding	4 months
11	83	f	Tuberculum sellae	Arterial hypertension, anticoagulant drug	4	Stroke	5 months
12	29	m	Convexity	−	3	Recurrence of anaplastic meningioma with intracerebral bleeding	11 months
13	87	f	Falx	Arterial hypertension, heart anomaly	3	Intraventricular bleeding	13 months
14	68	f	Tuberculum sellae	Arterial hypertension, anticoagulant drug	3	Pulmonary embolism	24 months
15	54	m	Intraventricular	Heart transplant, arterial hypertension, heart anomaly	3	Heart failure	4 years
16	51	m	Olfactory groove	Arterial hypertension, diabetes mellitus, depression	2	Heart failure	6 years and 3 months
17	74	f	Cerebellopontine angle	Heart insufficiency, diabetes mellitus, anticoagulant drug	3	Sepsis after aspiration pneumonia	7 years and 8 months
18	68	m	Sphenoid ridge	−	3	Stroke	10.10 years
19	65	f	Convexity	Asthma, heart insufficiency	3	Acute subdural hematoma	12 years
20	47	m	Sphenoid ridge	−	2	Recurrence of atypical meningioma	12.9 years
21	61	f	Cerebellopontine angle	Arterial hypertension, cancer, endocrine disease	3	Pneumonia after surgery of femur fracture	12.9 years

*COPD* chronic obstructive pulmonary disease

Multiple logistic regression analysis showed a significant impact of the ASA score (OR = 9.29, 95% CI [1.97; 43.62], p = 0.005) on mortality in elderly patients.

## Discussion

Meningiomas are the most common primary intracranial tumors in adults [1]. While surgery is the first treatment of choice for young patients, this is the subject of repeated discussions for the treatment of elderly patients, especially for patients with pre-existing comorbidities. In recent decades, many studies have explored the risk of meningioma surgery in elderly patients. Most of these studies focused only on elderly patients without including control groups of young patients [16–18]. On the other hand, the series presented in the literature comparing meningioma surgery in elderly and young patients contains small numbers of elderly patients, most of whom included fewer than one hundred elderly patients [7, 19–22]. Bateman et al. presented the largest series of patients with intracranial meningiomas in 2304 elderly and 6557 young patients from a nationwide inpatient sample database [6]. However, this series does not provide data about the neurological status of the patients or the characteristics of each patient’s tumor. We present here one of the largest single-center and representative series of 768 patients (484 young and 284 elderly patients) undergoing surgery for intracranial meningioma. To assess a particular age for a possible increased risk after meningioma surgery, the elderly cohort was subdivided into three subgroups, i.e., 65–69, 70–79, and over 80 years of age.

Most patients with intracranial meningiomas have tumor-related symptoms. In the literature, the number of asymptomatic patients in elderly and young cohorts ranges from 5 to 35.2% and 2 to 6.5%, respectively [18, 19]. In our cohort, only 9.5% of all the patients (10.3% of the young and 8.1% of the elderly patients) were asymptomatic. Interestingly, all patients over 80 years old were symptomatic. The tumors in most of our asymptomatic patients were usually space-occupying tumors, unclear tumors or suspicion of other tumors, such as metastases, especially in elderly patients with known carcinomas.

Despite differences such as age, number of pre-existing diseases and ASA score, even in elderly patients, high total resection rates were achieved in 79–88.6% of elderly patients compared to 62.9–90.3% of young patients [7, 18, 22, 23]. Li et al. reported a series of 70 patients over 65 years and 80 patients younger than 65 years with significantly low total resection rates of 50 and 68%, respectively [21]. In our cohort, we achieved a total resection rate of 84.5% in elderly patients (group II) and 76.2% in young patients (group I). The total resection rate was also high in the subgroups, but it decreased with advancing age: subgroup IIa 88.2%, IIb 82.6% and IIc 80.5%. The extent of tumor resection in our young cohort depended significantly on tumor size (r = 0.148, p = 0.007).

Morbidity after meningioma surgery has been reported in the literature for elderly and young patients at 7–69.6% and 8.8–57.5%, respectively [5–7, 19, 21]. Postoperative morbidities in our cohort were within this range, i.e., 37.2%, and was more common in elderly patients (46.5% in group II vs. 31.8% in group I). The three most common postoperative morbidities in the entire cohort were bleeding (12.9%), cranial nerve disorder (10%), and CSF fistula (8.1%). Postoperative bleeding, palsy, speech disorder, pneumonia and renal insufficiency were present more often in elderly patients. Patients over 80 years old had the highest bleeding rate (29.3%), and young patients had the lowest bleeding rate (9.1%). In elderly patients, males were more frequently affected by bleeding than females (r = 0.122, p = 0.001). Maurice-Williams and Kitchen reported a bleeding rate of 20% for elderly patients and no bleeding in young patients [24]. Cranial nerve disorder is one of the most common surgery-related complications reported in the literature [18, 21]. Postoperative bleeding, infections and deep vein thromboses were the three most common complications in elderly patients reported by Boviatsis et al. in a series of 108 elderly patients compared with 240 young patients [25].

Despite the higher rate of postoperative morbidities in elderly patients, many studies have shown an improvement in neurological status postoperatively [22, 24]. Zhao et al. demonstrated in their series of 115 elderly patients a significantly lower postoperative KPS (mean KPS of 79.6) than in 413 young patients (mean KPS 88.81) [26]. Li et al. showed no differences among elderly and young patients with a KPS of 90 or higher, 82.9 and 81.2%, respectively, in a follow-up of one year after surgery [21]. There are similar results in the series presented by Slot et al. [20]. The young and elderly patients (also subgroups IIa and IIb) in our cohort showed a significant improvement in postoperative KPS compared to preoperative KPS (p < 0.001). However, patients over 80 years old (subgroup IIc) showed deterioration in postoperative KPS compared to the preoperative KPS. The improvement in neurological conditions, even in the elderly, despite the higher rate of postoperative complications, can be explained by the fact that not all postoperative complications lead to persistent neurological disorders. Moreover, there was a further improvement in KPS at the last follow-up in all patients, even in patients over 80 years of age, compared to their postoperative KPS.

The mortality rate after meningioma surgery also varies greatly in the literature, ranging from 0 to 20% in elderly patients and 0–6% in young patients [5, 9, 19, 27–29]. Nakamura et al. and Li. et al. reported no death in their elderly and young cohort groups. However, in the cohort study by Nakamura et al., only 21 patients were included in the elderly group, and no patients with an ASA score of 4 or higher were included in the analysis [28]. Li et al. also investigated only patients with low ASA scores (ASA 1 and 2) in their cohort [21]. Brokinkel et al. showed a significant higher mortality rate in their elderly patients (22%) than in their young patients (6%). However, they did not see a significant differences of median overall survival in elderly patients after meningioma surgery compared with age- and sex-matched general population in Germany [30]. In our cohort, 21 patients [6 (1.2%) young and 15 (5.3%) elderly patients) died in an observation period of 17.4 years. Patients over 80 years old had the highest mortality rate (7 patients (17%)].

### Strengths and limitations of the study

To our Acknowledge, we present here one of the largest series of elderly and young patients with intracranial meningiomas in a single-center. All patient’s demographics, tumor and treatment characteristics, all postoperative complications within 30 days, and follow-up data were collected. As a result, it was possible to evaluate all relevant demographic and clinical data.

The limitation of the study is the retrospective study design. Another limitation of the study is that tumor size and brain edema were not determined volumetrically. However, the tow parameters were not primary points of our investigation and most published studies on this issue do not provide any information about tumor and brain edema volume. The volumetric assessment of tumor size and brain edema will be a focus of investigation in the future.

## Conclusions

Our study demonstrates that meningioma surgery is associated with a higher rate of postoperative complications in elderly patients than in young patients. With the exception of patients over 80 years of age, a significant improvement in neurological status postoperatively was found in our cohort. In addition, the KPS at the last follow-up improved in all patients, even in those over 80 years old. Therefore, based on our data, we conclude that all symptomatic patients who have an indication for surgical therapy should be treated. Patients in high risk groups, such as those over 80 years old and those with multiple comorbidities and high operative risk, should be consulted and supervised perioperatively by an experienced team of neurosurgeons and anesthesiologists.

## Supplementary Information

Below is the link to the electronic supplementary material.

Supplementary material 1 (DOCX 29.7 kb)

## Data Availability

All data relating to this research project are available at the corresponding author can be viewed at any time if desired.
