# Supplementary material for: Risks and neurological benefits of meningioma surgery in elderly patients compared to young patients
Source: J Neurooncol. 2021 Sep 1;154(3):335–44. doi: 10.1007/s11060-021-03832-5 (PMC8484216; doi:10.1007/s11060-021-03832-5)
Supplement: Supplementary file 1 — Supplementary material 1 (DOCX 29.7 kb) [file 11060_2021_3832_MOESM1_ESM.docx]

**Supplemental Table**: KPS preoperative, postoperative and at the time of last follow-up.

| **KPS preoperative, postoperative and at last follow-up** | **All patients** | | **Group I**  **(age: ≤ 64 yrs)** | | | **Group II**  **(age: ≥ 65 yrs)** | | | | **Subgroup IIa**  **(age: 65-69 yrs)** | | | **Subgroup IIb**  **(age: 70-79 yrs)** | | | **Subgroup IIc**  **(age: ≥ 80 yrs)** | | |
| --- | --- | --- | --- | --- | --- | --- | --- | --- | --- | --- | --- | --- | --- | --- | --- | --- | --- | --- |
|  | **No** | **%** |  | **No** | **%** |  | **No** | **%** |  | **No** | **%** |  | **No** | **%** |  | **No** | **%** |  |
|  |  |  |  |  |  |  |  |  |  |  |  |  |  |  |  |  |  |  |
| **Preoperative KPS** |  |  |  |  |  |  |  |  |  |  |  |  |  |  |  |  |  |  |
|  |  |  |  |  |  |  |  |  |  |  |  |  |  |  |  |  |  |  |
| **mean** | 79.90 | SD±12.31 |  | 81.44 | SD±11.11 |  | 77.28 | SD±13.74 |  | 79.89 | SD±11.70 |  | 77.76 | SD±14.50 |  | 70.48 | SD±14.13 |  |
|  |  |  |  |  |  |  |  |  |  |  |  |  |  |  |  |  |  |  |
| 10 | - | - |  | - | - |  | - | - |  | - | - |  | - | - |  | - | - |  |
| 20 | 5 | 0.7 |  | 2 | 0.4 |  | 3 | 1.1 |  | - | - |  | 3 | 2.1 |  | - | - |  |
| 30 | 3 | 0.4 |  | 1 | 0.2 |  | 2 | 0.7 |  | 1 | 1 |  | - | - |  | 1 | 2.4 |  |
| 40 | - | - |  | - | - |  | - | - |  | - | - |  | - | - |  | - | - |  |
| 50 | 14 | 1.8 |  | 4 | 0.8 |  | 10 | 3.5 |  | 2 | 2 |  | 3 | 2.1 |  | 5 | 12.2 |  |
| 60 | 42 | 5.5 |  | 18 | 3.7 |  | 24 | 8.5 |  | 2 | 2 |  | 14 | 9.7 |  | 8 | 19.5 |  |
| 70 | 159 | 20.7 |  | 89 | 18.4 |  | 70 | 24.6 |  | 25 | 24.8 |  | 36 | 25 |  | 9 | 22 |  |
| 80 | 272 | 35.4 |  | 184 | 38 |  | 88 | 31 |  | 38 | 37.6 |  | 38 | 26.4 |  | 12 | 29.3 |  |
| 90 | 205 | 26.7 |  | 139 | 28.7 |  | 66 | 23.2 |  | 21 | 20.8 |  | 39 | 27.1 |  | 6 | 14.6 |  |
| 100 | 59 | 7.7 |  | 42 | 8,7 |  | 17 | 6 |  | 9 | 8.9 |  | 10 | 6.9 |  | - | - |  |
| **NK** | 9 | 1.2 |  | 5 | 1 |  | 4 | 1.4 |  | 3 | 3 |  | 1 | 0.7 |  | - | - |  |
|  |  |  |  |  |  |  |  |  |  |  |  |  |  |  |  |  |  |  |
|  |  |  |  |  |  |  |  |  |  |  |  |  |  |  |  |  |  |  |
| **Postoperative KPS** |  |  |  |  |  |  |  |  |  |  |  |  |  |  |  |  |  |  |
|  |  |  |  |  |  |  |  |  |  |  |  |  |  |  |  |  |  |  |
| **mean** | 83.47 | SD±15.46 |  | 85.36 | SD±12.29 |  | 80.21 | SD±19.35 |  | 83.98 | SD±14.34 |  | 81.95 | SD±16.66 |  | 65.75 | SD±30.11 |  |
|  |  |  |  |  |  |  |  |  |  |  |  |  |  |  |  |  |  |  |
| 0 | 8 | 1 |  | 1 | 0.2 |  | 7 | 2.5 |  | 1 | 1 |  | 2 | 1.4 |  | 4 | 9.8 |  |
| 10 | - | - |  | - | - |  | - | - |  | - | - |  | - | - |  | - | - |  |
| 20 | 6 | 0.8 |  | 2 | 0.4 |  | 4 | 1.4 |  | - | - |  | 2 | 1.4 |  | 2 | 4.9 |  |
| 30 | 2 | 0.3 |  | 1 | 0.2 |  | 1 | 0.4 |  | - | - |  | - | - |  | 1 | 2.4 |  |
| 40 | 3 | 0.4 |  | 1 | 0.2 |  | 2 | 0.7 |  | - | - |  | 1 | 0.7 |  | 1 | 2.4 |  |
| 50 | 9 | 1.2 |  | 2 | 0.4 |  | 7 | 2.5 |  | 1 | 1 |  | 2 | 1.4 |  | 4 | 9.8 |  |
| 60 | 27 | 3.5 |  | 15 | 3.1 |  | 12 | 4.2 |  | 4 | 4 |  | 7 | 4.9 |  | 1 | 2.4 |  |
| 70 | 92 | 12 |  | 57 | 11.8 |  | 35 | 12.3 |  | 18 | 17.8 |  | 12 | 8.3 |  | 5 | 12.2 |  |
| 80 | 174 | 22.7 |  | 108 | 22.3 |  | 66 | 23.2 |  | 14 | 13.9 |  | 42 | 29.2 |  | 10 | 24.4 |  |
| 90 | 319 | 41.5 |  | 208 | 43 |  | 111 | 39.1 |  | 44 | 43.6 |  | 58 | 40.3 |  | 9 | 22 |  |
| 100 | 120 | 15.6 |  | 86 | 17.8 |  | 34 | 12 |  | 16 | 15.2 |  | 17 | 11.8 |  | 3 | 7.3 |  |
| **NK** | 8 | 1 |  | 3 | 0.6 |  | 5 | 1.8 |  | 3 | 3 |  | 1 | 0.7 |  | 1 | 2.4 |  |
| **KPS at last Follow-up** |  |  |  |  |  |  |  |  |  |  |  |  |  |  |  |  |  |  |
|  |  |  |  |  |  |  |  |  |  |  |  |  |  |  |  |  |  |  |
| **mean** | 88.15 | SD±15.60 |  | 89.18 | SD±13.27 |  | 86.04 | SD±19.39 |  | 87 | SD±19.05 |  | 87.63 | SD±14.95 |  | 73.33 | SD±35.81 |  |
|  |  |  |  |  |  |  |  |  |  |  |  |  |  |  |  |  |  |  |
| 0 | 13 | 1.7 |  | 5 | 1 |  | 8 | 2.8 |  | 3 | 3 |  | 2 | 1.4 |  | 3 | 7.3 |  |
| 10 | - | - |  | - | - |  | - | - |  | - | - |  | - | - |  | - | - |  |
| 20 | - | - |  | - | - |  | - | - |  | - | - |  | - | - |  | - | - |  |
| 30 | - | - |  | - | - |  | - | - |  | - | - |  | - | - |  | - | - |  |
| 40 | 1 | 0.1 |  | 1 | 0.2 |  | - | - |  | - | - |  | - | - |  | - | - |  |
| 50 | 2 | 0.3 |  | - | - |  | 2 | 0.7 |  | - | - |  | 1 | 0.7 |  | 1 | 2.4 |  |
| 60 | 9 | 1.2 |  | 6 | 1.2 |  | 3 | 1.1 |  | - | - |  | 3 | 2.1 |  | - | - |  |
| 70 | 31 | 4 |  | 22 | 4.5 |  | 9 | 3.2 |  | 6 | 5.9 |  | 2 | 1.4 |  | 1 | 2.4 |  |
| 80 | 80 | 10.4 |  | 54 | 11.2 |  | 26 | 9.2 |  | 6 | 5.9 |  | 19 | 13.2 |  | 1 | 2.4 |  |
| 90 | 322 | 41.9 |  | 210 | 43.4 |  | 112 | 39.4 |  | 44 | 43.6 |  | 60 | 41.7 |  | 8 | 19.5 |  |
| 100 | 181 | 23.6 |  | 131 | 27.1 |  | 50 | 17.6 |  | 21 | 20.8 |  | 27 | 18.8 |  | 4 | 9.8 |  |
| NK | 129 | 16.8 |  | 55 | 11.4 |  | 74 | 26.1 |  | 21 | 20.8 |  | 30 | 20.8 |  | 23 | 56.1 |  |
|  |  |  |  |  |  |  |  |  |  |  |  |  |  |  |  |  |  |  |

Note: NK= not known; yrs= years.
